# Social conformism and confidence in systems as additional psychological antecedents of vaccination: a survey to explain intention for COVID-19 vaccination among healthcare and welfare sector workers, France, December 2020 to February 2021

**DOI:** 10.2807/1560-7917.ES.2022.27.17.2100617

**Published:** 2022-04-28

**Authors:** Simi Moirangthem, Cyril Olivier, Amandine Gagneux-Brunon, Gérard Péllissier, Dominique Abiteboul, Isabelle Bonmarin, Elisabeth Rouveix, Elisabeth Botelho-Nevers, Judith E Mueller

**Affiliations:** 1 EHESP French School of Public Health, Paris and Rennes, France; 2 Research Group for the Prevention of Occupational Infections in Healthcare Workers (GERES), Paris, France; 3 Chaire PreVacCI de l'Institut Presage, Université Jean Monnet Saint-Etienne, Saint-Étienne, France; 4 CIC-1408, Vaccinologie, INSERM, CHU St Etienne, Saint-Étienne, France; 5 Santé Publique France, Saint-Maurice, France; 6 Université Versailles Saint Quentin en Yvelines, APHP, CHU Ambroise Paré, Versailles, France; 7 Institut Pasteur, Paris, France

**Keywords:** COVID-19 vaccination, healthcare workers, vaccine hesitancy, vaccine acceptance

## Abstract

**Background:**

The start of the COVID-19 vaccination campaign among French healthcare and welfare sector workers in January 2021 offered an opportunity to study psychological antecedents of vaccination in this group.

**Aim:**

We explored whether knowledge and attitude items related to social conformism and confidence in systems contributed to explaining intention for COVID-19 vaccination.

**Methods:**

We developed a knowledge and attitude questionnaire with 30 items related to five established and two hypothetical psychological antecedents of vaccination (KA-7C). The online questionnaire was distributed from 18 December 2020 to 1 February 2021 through chain-referral via professional networks, yielding a convenience sample. We used multivariable logistic regression to explore the associations of individual and grouped KA-7C items with COVID-19 vaccine intention.

**Results:**

Among 5,234 participants, the vaccine intention model fit (pseudo R-squared values) increased slightly but significantly from 0.62 to 0.65 when adding social conformism and confidence in systems items. Intention to vaccinate was associated with the majority opinion among family and friends (OR: 11.57; 95% confidence interval (CI): 4.51–29.67) and a positive perception of employer’s encouragement to get vaccinated (vs negative; OR: 6.41; 95% CI: 3.36–12.22). The strongest association of a knowledge item was identifying the statement ‘*Some stages of vaccine development (testing) have been skipped because of the epidemic emergency.’* as false (OR: 2.36; 95% CI: 1.73–3.22).

**Conclusion:**

The results suggest that social conformism and confidence in systems are distinct antecedents of vaccination among healthcare and welfare workers, which should be taken into account in vaccine promotion.

## Introduction

Vaccination is one of the main tools to respond to the current coronavirus disease (COVID-19) pandemic. Healthcare workers (HCWs) are among the priority groups in most countries who aim to provide them with protection given their continuous exposure, protect the healthcare system from absenteeism and prevent nosocomial transmission of severe acute respiratory syndrome coronavirus 2 (SARS-CoV-2) [1]. In France, COVID-19 vaccination of HCWs has been recommended from early January 2021 on, initially limited to those aged 50 years or older or with underlying conditions, and without any limitations from early February 2021 on.

In July 2021, the COVID-19 vaccination coverage for at least one dose among HCWs in France was estimated at 60.5% and 80.5% in nursing homes and in private practices, respectively [2]. At the same time, a strong gradient of the vaccination rates from medical professions to nurses and nurse assistants was described in hospitals, similar to the expressed intentions in surveys performed France in 2020 [3,4]. A COVID-19 vaccine mandate for healthcare and welfare sector workers entered into force in France on 15 September 2021 and includes since 30 January 2022 a booster dose. To prepare a long term strategy of COVID-19 vaccine promotion, it will be important to understand and follow up antecedents of COVID-19 vaccine acceptance.

The term vaccine hesitancy was coined to describe the attitude of delay in acceptance or refusal towards vaccination despite availability [5]. To better understand the source of vaccine hesitancy and to evaluate interventions to mitigate, it is important to consider the psychological aspects of human behaviour and choice. The original 3C psychological antecedents model [5] included (i) confidence i.e. trust in vaccines, the system that delivers them and motivations of policy makers who decide on needed vaccines; (ii) complacency i.e. need of the vaccine given its effectiveness and severity of the disease; and (iii) convenience i.e. accessibility [5]. Betsch et al. proposed an expanded 5C-scale including two additional antecedents: (i) calculation (deliberation on risks and benefits); and (ii) collective responsibility (sense of altruism towards getting vaccinated) [6]. In our study we explore whether items related to two additional antecedents can improve the explanation of vaccine intention. First, we propose adding social conformism as a psychological antecedent. Taking decisions by imitating peers is known to be an important heuristic that helps to reduce mental load in daily life [7]. In several discrete choice experiments higher theoretical vaccination acceptance was found in scenarios presenting higher coverage in the community [8-10]. Furthermore, we examine whether the confidence psychological antecedent should discriminate between confidence in the vaccine and vaccine-related system and confidence in the wider circle of systems, including authorities and employer. In a study looking at French-speaking general practitioners in late 2020, the distrust in the Ministry of Health and in vaccine safety appeared to lead to lower COVID-19 vaccination acceptance [11]. Additionally, as pointed out by Larson et al., confidence can be separated into product trust, provider trust and political or system trust [12].

The roll-out of COVID-19 vaccination among HCWs in France provided an opportunity to study whether knowledge and attitude items related to social conformism and confidence in systems (authorities and employer) contributed to explaining the intention for COVID-19 vaccination among healthcare and welfare sector workers.

## Methods

### Participant recruitment

From 18 December 2020 to 1 February 2021, the Research Group for the Prevention of Occupational Infections in Healthcare Workers published an online survey via the Sphinx online survey platform. This was disseminated by email chain-referral throughout France, including overseas departments. Several formal and informal networks of hospital-based and private practice HCWs and of nursing home directors contributed to its dissemination. Since each participant could forward the survey across their own network, we did not estimate a response rate; nor were visits to the survey website counted.

### Data collection

The questionnaire consisted of three parts. The first and third part collected socio-demographic, professional and health-related characteristics of the participants, and information on the intention to accept and recommend COVID-19 vaccination. The second part of the survey directed participants, by choosing a shape (square or triangle), to either a discrete choice experiment [13] or to the present questionnaire on knowledge and attitudes. Effective survey completion time was ca 8 min.

### Questionnaire development

The knowledge and attitude (KA) questionnaire was based on the 5C-scale for evaluation of psychological antecedents presented by Betsch et al. [5]. Since the 5C-scale relates to vaccination in general, we adapted questions to apply specifically to COVID-19 vaccination of healthcare and welfare sector workers in the epidemic context in France. Item groups related to the two additional antecedents were included in the KA-7C questionnaire: social conformism and confidence in systems (authorities and employer). One author developed a draft of the KA-7C questionnaire which was reviewed by other co-authors for coherence with the 5C-scale. Each item group consisted of at least one attitude and knowledge question. In total, the KA-7C questionnaire had 30 questions: nine questions were associated with the attitude towards the vaccine and systems; 19 questions were associated with the knowledge about the vaccines, their development and COVID-19. A 5-point Likert scale was used to simplify questionnaire administration. Two items were general attitude questions on confidence in the authorities for managing the public health and economic crisis caused by COVID-19 and concern about the COVID-19 epidemic, both on an 11-point scale (Supplementary Table S1). Knowledge items were either presented as a statement to which participants could answer ‘*Right’*, ‘*Do not know’* or ‘*Wrong’*, or requested a single choice answer to a question from several options which included ‘*Do not know’* (Supplementary Table S1).

Prior to finalisation, the questionnaire was reviewed by occupational health specialists in hospitals who are in charge of vaccine promotion towards HCWs, and pilot-tested in think-aloud sessions with HCWs including physicians, pharmacists and nurses.

### Data analysis

Knowledge variables were coded as incorrect answer, ‘*Do not know’* and correct answer. We kept ‘*Do not know’* as a distinct modality of the knowledge variables, to distinguish the specific situation of participants recognising their lack of knowledge. Answers to the general attitude questions (i.e on confidence in crisis management and concern about the COVID-19 epidemic) were transformed into three categories: (i) low: 0–3; (ii) medium: 4–6; and (iii) high: 7–10. Other attitude items were maintained on a 5-point scale.

We used bivariate logistic regression models to explore the association of participant characteristics and individual KA-7C items with vaccine intention. Initial analyses explored vaccine intention as ‘*Yes’* with and without the *’Do not know’* modality. No major differences were detected between these analyses, therefore the final analyses were carried out on the variable *’Yes’* vs *’No/Do not know’* to clearly focus on vaccine intention*.* We created a variable for the different periods of survey participation: (i) period 1 from 18 December 2020 to 4 January 2021, which was the early phase of the COVID-19 vaccination campaign targeting nursing home residents; (ii) period 2 from 5 January to 14 January 2021, when vaccination was expanded to HCWs aged 50 years or older; and (iii) period 3 from 15 January to 1 February 2021, when the COVID-19 vaccination campaign was expanded to the general population aged 75 years or older or to people having specific high-risk comorbidities such as rare immune disorders.

We evaluated collinearity between the KA-7C items using the collin command in Stata version 16.1 (StataCorp, College Station, Texas, United States (US)). For variables with variance inflation factor > 2, we conducted pairwise Spearman correlation testing and considered any correlation with rho < 0.70 as not critical. To identify socio-demographic and health-related determinants of vaccine intention, we included variables with p value < 0.20 in bivariate regression into a multivariable logistic regression model using a stepwise forward procedure (basic model). In France, most professional categories in the healthcare and welfare sector are well-defined and correlate with educational trajectories [14,15]. We therefore did not include educational level in the models.

**Figure 1:**
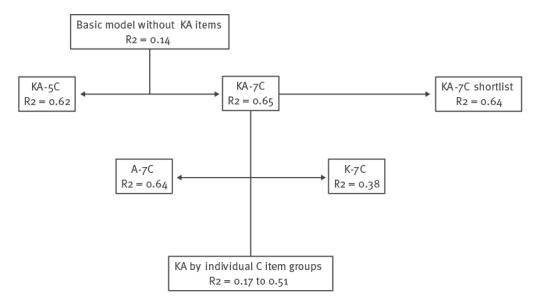
Flowchart of model examination to explore the fit of regression models with KA-7C items explaining vaccine intention at the start of the COVID-19 vaccination campaign, France, 18 December 2020–1 February 2021

We examined the contribution of items and item groups to the explanation of vaccine intention variation based on MacFadden pseudo R-squared values (R2) where values above 0.20–0.40 indicate excellent fit. The significance of the contribution of the hypothetical antecedent item groups was assessed based on the nested log likelihood ratio test. Figure 1 presents: a basic model adjusting only for socio-demographic variables; a model with all 30 KA-7C items compared with a model limited to five antecedents (5C); models including only knowledge compared with only attitude items and models with individual C-item groups.

A: attitude item; COVID-19: coronavirus disease; K: knowledge item; R2: pseudo R-squared value.

5C and 7C refer to the five established (5C) and additional two hypothetical antecedents (7C) of vaccination respresented by item group.

Finally, for presentation of effect estimates, we defined a shortlist of 14 KA-7C items, selecting for each 7C item group the attitude and the knowledge item with the highest pseudo R-squared value. We estimated the association of each KA-7C item and item group with vaccine intention in multivariable models reporting odds ratios (OR) and 95% confidence intervals (CI). All models controlled for age group, sex, professional category, work in a nursing home and period of study participation.

## Results

### Participants

The survey reached all French regions, including the overseas departments, although participation in the latter was sporadic. A total of 9,580 participants from diverse health-related careers and sectors participated. The KA-7C questionnaire was completed by 5,234 participants, with similar distribution across the periods defined by roll-out of the vaccination campaign: 38.7%, 30.9% and 30.4%. Women represented 78.4% of participants and 23.2%, 40.0% and 36.8%, respectively, were aged 18–34 years, 35–49 years and 50 years or older (Table). Nurses represented 22.9%, nurse assistants 9.4%, biomedical professionals (including physicians, midwives, pharmacists and biologists) 27.7%, other paramedical staff 15.7% and administration staff 24.4% (Table). Among physicians in our sample, 59% were female and median age group was 35–49 years (cf.d with 50% and 49.3 years mean age according to official estimates in 2021 [16]). Among nurses, 85% were female and median age group was 35–49 years (cf.d with 88% and 40.2 years mean age in 2011 [17]).

**Table:** Survey responses by healthcare and welfare sector workers at the start of the COVID-19 vaccination campaign, France, 18 December 2020–1 February 2021 (n = 5,234)

Characteristics and shortlist of KA-7C items		n	%	Intention to get COVID-19 vaccination				Full multivariable model^a^		
				No/DNK		Yes		Yes vs No/DNK		
				n	%	n	%	OR		95% CI
**Socio-demographic characteristics**										
Age (years)	18–34	1,215	23.2	681	56.1	534	44.0	Ref		
	35–49	2,092	40.0	932	44.6	1,160	55.5	1.04		0.80–1.35
	≥ 50	1,927	36.8	578	30.0	1,349	70.0	1.47		1.11–1.96
Sex	Female	4,103	78.4	1,889	46.0	2,214	54.0	Ref		
	Male	1,131	21.6	302	26.7	829	73.3	1.22		0.94–1.60
Profession	Nurses	1,197	22.9	603	50.4	594	49.6	Ref		
	Nurse assistants	491	9.4	341	69.5	150	30.6	0.78		0.51–1.19
	Other paramedical staff^b^	819	15.7	407	49.7	412	50.3	0.73		0.53–1.01
	Biomedical professionals^c^	1,449	27.7	287	19.8	1,162	80.2	1.25		0.92–1.70
	Admin/technical staff	1,278	24.4	553	43.3	725	56.7	1.03		0.77–1.37
Work in a nursing home	No	4,429	84.6	1,766	39.9	2,663	60.1	Ref		
	Yes	805	15.4	425	52.8	380	47.2	0.97		0.72–1.31
Study period	1	2,026	38.7	1,113	54.9	913	45.1	Ref		
	2	1,618	30.9	574	35.5	1,044	64.5	1.73		1.34–2.23
	3	1,590	30.4	504	31.7	1,086	68.3	2.20		1.68–2.88
**Confidence in COVID-19 vaccine**										
*’I am afraid of having a severe side effect of vaccination.’*	Strongly disagree	1,203	23.0	127	10.6	1,076	89.4	12.36	7.76–19.70	
	Disagree	1,341	25.6	245	18.3	1,096	81.7	10.52	7.02–15.79	
	Undecided	959	18.3	418	43.6	541	56.4	4.87	3.30–7.17	
	Agree	891	17.0	652	73.2	239	26.8	2.19	1.48–3.24	
	Strongly agree	840	16.1	749	89.2	91	10.8	Ref		
*’The security of vaccines is monitored not only at the national level, but also in collaboration between European countries.’*	False (i)	92	1.76	79	85.9	13	14.1	Ref		
	DNK	855	16.3	596	69.7	259	30.3	1.43	0.42–4.84	
	True (c)	4,287	81.9	1,516	35.4	2,771	64.6	2.20	0.66–7.29	
**Confidence in systems**										
*’If my employer encourages me to get vaccinated, this…’*	Dissuades me	274	5.2	247	90.2	27	9.9	Ref		
	Has no effect	3,409	65.1	1,695	49.7	1,714	50.3	2.71	1.45–5.06	
	Motivates me	1,551	29.6	249	16.1	1,302	84.0	6.41	3.36–12.22	
*’Some stages of vaccine development (testing) have been skipped because of the epidemic emergency.’*	False (c)	2,252	43.0	399	17.7	1,853	82.3	2.36	1.73–3.22	
	DNK	2,023	38.7	1,071	52.9	952	47.1	2.02	1.50–2.71	
	True (i)	959	18.3	721	75.2	238	24.8	Ref		
**Complacency**										
*’I am afraid of getting a severe form of COVID-19.’*	Strongly disagree	1,109	21.2	528	47.6	581	52.4	Ref		
	Disagree	1,524	29.1	673	44.2	851	55.8	1.28	0.94–1.73	
	Undecided	1,222	23.4	488	39.9	734	60.1	1.38	0.96–1.93	
	Agree	796	15.2	284	35.7	512	64.3	1.88	1.30–2.71	
	Strongly agree	583	11.1	218	37.4	365	62.6	2.76	1.76–4.33	
*’The gravity of the epidemic requires making vaccines quickly available.’*	False (i)	411	7.9	331	80.5	80	19.5	Ref		
	DNK	513	9.8	387	75.4	126	24.6	1.73	0.97–3.12	
	True (c)	4,310	82.4	1,473	34.2	2,837	65.8	1.72	1.05–2.82	
**Convenience**										
*’In practice, it will be difficult for me to get vaccinated.’*	Strongly disagree	2,429	46.4	772	31.8	1,657	68.2	Ref		
	Disagree	1,386	26.5	610	44.0	776	56.0	0.93	0.72–1.20	
	Undecided	765	14.6	436	57.0	329	43.0	0.60	0.44–0.81	
	Agree	361	6.9	182	50.4	179	49.6	1.08	0.71–1.65	
	Strongly agree	293	5.6	191	65.2	102	34.8	0.71	0.41–1.22	
*‘It is necessary to have two injections to be immunised.’* ^d^	False (i)	159	3.0	98	61.6	61	38.4	Ref		
	DNK	524	10.0	372	71.0	152	29.0	0.76	0.38–1.51	
	True (c)	4,551	87.0	1,721	37.8	2,830	62.2	1.14	0.62–2.09	
**Calculation**										
*’I think that vaccination against COVID-19 will have more benefits than risks for me.’*	Strongly disagree	496	9.5	437	88.1	59	11.9	Ref		
	Disagree	670	12.8	603	99.0	67	10.0	0.74	0.42–1.31	
	Undecided	1,136	21.7	841	74.0	295	26.0	1.33	0.80–2.20	
	Agree	1,205	23.0	242	20.1	963	79.9	6.39	3.82–10.67	
	Strongly agree	1,727	33.0	68	3.9	1,659	96.1	16.97	9.78–29.47	
*‘For a person with risk factors, these vaccines have more benefits than risks in the current epidemic situation.’* ^d^	False (i)	148	2.8	124	83.8	24	16.2	Ref		
	DNK	875	16.7	700	80.0	175	20.0	0.76	0.32–1.81	
	True (c)	4,211	80.5	1,367	32.5	2,844	67.5	0.87	0.37–2.00	
**Collective responsibility**										
*’Getting vaccinated will also be a collective action to stop the crisis caused by the epidemic.’*	Strongly disagree	253	4.8	231	91.3	22	8.7	Ref		
	Disagree	318	6.1	297	93.4	21	6.6	0.70	0.28–1.73	
	Undecided	686	13.1	620	90.4	66	9.6	0.71	0.33–1.55	
	Agree	1,222	23.4	612	50.1	610	49.9	2.35	1.12–4.93	
	Strongly agree	2,755	52.6	431	15.6	2,324	84.4	5.04	2.44–10.43	
*‘The vaccine blocks transmission of the virus to those around you in case of infection.’* ^d^	False (c)	781	14.9	1095	41.7	1,531	58.3	0.91	0.67–1.22	
	DNK	1,827	34.9	820	44.9	1,007	55.1	0.95	0.69–1.30	
	True (i)	2,626	50.2	276	35.3	505	64.7	Ref		
**Social conformism**										
*‘Among your family and friends, how would you describe the majority opinion towards COVID-19 vaccination?’*	Very favourable	390	7.5	8	2.1	382	98.0	11.57	4.51–29.67	
	Favourable	1,418	27.1	199	14.0	1,219	86.0	4.42	2.70–7.22	
	Both skeptical and favourable	1,653	31.6	701	42.4	952	57.6	2.28	1.43–3.63	
	Skeptical	1,319	25.2	897	68.0	422	32.0	1.59	0.99–2.56	
	Very skeptical	454	8.7	386	85.0	68	15.0	Ref		
*‘Do you know the approximate percentage of healthcare workers who intend to get the COVID-19 vaccine?’*	30% (i)	1,743	33.3	906	52.0	837	48.0	Ref		
	DNK	2,064	39.4	937	45.4	1,127	54.6	1.14	0.89–1.46	
	60% and 90% (c)	1,427	27.3	348	24.4	1,079	75.6	1.41	1.07–1.86	

COVID-19: coronavirus disease; DNK: Do not know; OR: odds ratio; 95% CI: 95% confidence intervals.

(c): correct knowledge item response; (i): incorrect knowledge item response.

^a^ Full multivariable model with shortlist KA-7C items adjusting for age group, sex, professional category, work in a nursing home and period of study participation.

^b^ Includes workers in direct contact with patients, except biomedical professions (see below), nurses and nurse-assistants: e.g., physiotherapists, dieticians, psychologists and educators.

^c^ Includes physicians, midwives, dentists, pharmacists and biologists.

^d^ These questions were introduced as follows: ‘*For the most advanced COVID-19 vaccines (close to licensure), the scientific data show that …’.*

Working at least part-time in a nursing home was reported by 805 (15.4%) participants (Table). Three-thousand and thirty-four participants (58.1%) indicated vaccine intention against COVID-19, 1,153 (22.0%) indicated no intention, while 1,038 (19.8%) did not know yet. Among participants, 2,779 (53.1%) reported vaccination against influenza during the 2019/20 winter season. The variable on receiving the previous influenza vaccine in 2019/20 was highly associated with COVID-19 vaccine intention but not included in models to avoid overfitting (data not shown).

### Exploration of model fit

Compared with a model including the initial 5C item groups, the addition of confidence in systems and social conformism increased the model fit slightly but significantly, from R2 = 0.62 to 0.65 (p < 0.001) (Figure 1). The model with attitude 7C-items only had a substantially higher R2 when compared with the model with knowledge 7C-items only (0.64 vs 0.38).

**Figure 2:**
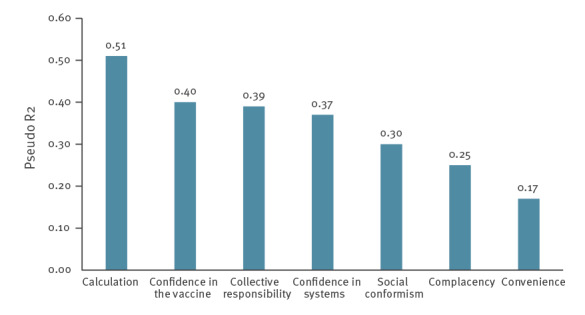
Fit of regression models explaining vaccination intention among healthcare and welfare sector workers at the start of the COVID-19 vaccination campaign, contribution by each 7C-item group, France, 18 December 2020–1 February, 2021 (n = 5,234)

Model fits (R2), corresponding to the percentage of variation in vaccine intention that can be explained, ranged from 0.17 to 0.51 for individual item groups (Figure 2). Confidence in systems and social conformism showed an R2 of 0.37 and 0.30, respectively.

Pseudo R-squared (R2) values obtained from multivariable regression models including vaccine intention and items from a given C item group, adjusting for age group, sex, professional category, work in a nursing home and period of study participation.

**Figure 3:**
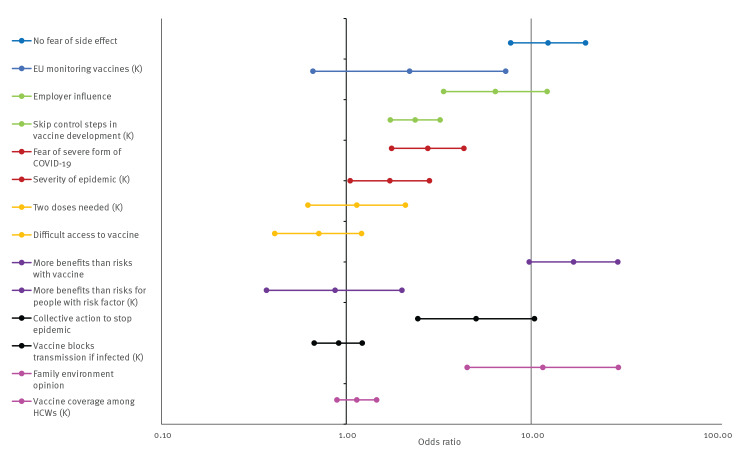
Associations with COVID-19 vaccination intention among healthcare and welfare sector workers, France, 18 December 2020–1 February 2021 (n = 5,234) at the start of the COVID-19 vaccination campaign

COVID-19: coronavisus disease; EU: European Union; HCWs: healthcare workers; K: knowledge variable.

^a^ Points and bars represent odds ratios and 95% confidence intervals estimated in a multivariable model adjusting age group, sex, professional category, work in a nursing home and study phase and including all KA-7C items of the questionnaire shortlist.

^b^ Only highest vs reference categories are shown.

In a full model that included the shortlist KA-7C items and adjusted for socio-demographic characteristics (Supplementary Table S2 showing results of all KA-7C items), the strongest associations were observed for a positive attitude regarding the vaccine’s benefit-risk balance (strongly agree vs strongly disagree, OR: 16.81; 95% CI: 9.66–29.25), fear of a severe side effect (strongly agree vs strongly disagree, OR: 12.47; 95% CI: 7.80–19.92) and a very favourable majority opinion among family and friends (vs very skeptical, OR: 11.02; 95% CI: 4.19–29.01) (Table, Figure 3). The strongest association with a knowledge item was identifying the statement *’Some stages of vaccine development (testing) have been skipped because of the epidemic emergency.’* as being false (OR: 2.36; 95% CI: 1.73–3.22).

## Discussion

In this cross-sectional study of a convenience sample of French healthcare and welfare sector workers exploring the psychological antecedents of COVID-19 vaccination, we found that items referring to social conformism and confidence in systems contributed to the explanation of vaccine intention, in addition to the 5C-model previously presented by Betsch et al. [5]. While the additional explanatory effect of including the two additional item groups was significant but relatively small, the effect sizes of corresponding items (opinion in private environment and perception of employer’s encouragement) were among the strongest in multivariable analysis.

While the KA-7C questionnaire explained 65% of the variation in vaccine intention, most explanatory power came from attitude items, contrasting with limited contribution from knowledge items. Regarding social conformism, the response to the question about majority opinion on COVID-19 vaccination among colleagues or family and friends was strongly associated with vaccine intention of the individual healthcare and welfare sector worker. Vaccination is a socially influenced process, and given the tendency towards homophily (self-selected association with similar people) [18], those who intend to vaccinate are likely to be in a social network with people who share the same sentiments and vice versa [19]. Our results are in concordance with results of previous discrete choice experiments, where the presentation of higher levels of vaccine coverage in the community was associated with greater theoretical vaccine acceptance among HCWs (seasonal influenza and pertussis vaccines) [9], adolescents (human papilloma virus vaccine) [20] and university students (measles and meningococcal vaccines) [8]. The heuristic concept of imitating-your-peers [7] should be further explored in promotion of COVID-19 and other vaccines towards healthcare and welfare sector workers. Taking into account local cultures and group norms, creating chain effects within social networks should help normalise vaccination. Research on vaccine acceptance should therefore increasingly target specific milieus, professional categories and social networks. Any interventions will require a good understanding of the positive or negative social influences acting within the target group.

During the COVID-19 epidemic, confidence in national authorities has become an important polarising characteristic [19] that affects adherence to epidemic control measures and vaccine intention in populations. We addressed this aspect in two ways, by asking questions about participants’ confidence in authorities with regard to COVID-19 crisis management and their perception of a vaccine recommendation from the employer. In France, having previously voted for political parties on the far-left or far-right spectrum, was negatively associated with early COVID-19 vaccine intention in spring 2020 [19]; and a negative perception of healthcare working conditions has been found to be inversely related to influenza vaccine uptake [21]. HCWs play a crucial role at the interface between public health officials and the general population. However, many HCWs are not vaccinology experts and are well aware of how little they know of various vaccines and their inability to answer some of their patients’ questions [22]. As stated by Ward et al., the relationship between public health authorities and HCWs in France has deteriorated over the past 30 years along with depleted funding for public hospitals and the restructuring of the health system [23]. Neither change has helped to induce a positive perception of the vaccine-related or wider systems.

In France, only authorities can issue mandates and often mandates are expected from authorities, which explains why vaccination is highly politicised. Vaccine mandates for HCWs, often supported by hospital managers and doctors, can be seen as either the solution or as an aggravating factor to the problem of suboptimal vaccine coverage among HCWs. Primary and booster vaccination against COVID-19 has become mandatory for healthcare and welfare sector workers in France, and contract terminations for non-compliance with the mandate have been reported since October 2021. Further research is needed to evaluate in how far general, not vaccine-related, societal trust should be taken into account as a separate psychological antecedent of acceptance of other recommended vaccines and for the general population.

Knowledge items played a small role in explaining COVID-19 vaccine intention. The healthcare and welfare sector workers in our sample were a heterogeneous group with education ranging from vocational training to over 6 years of medical training. The frequently observed difference in influenza vaccine uptake between professional categories has led to the conclusion that professionals with shorter educational duration need more or better information on vaccines. Previous vaccine promotion campaigns among French HCWs have focused on organising meetings to deliver scientific messages and answer any questions as decision makers tend to think that lack of knowledge might be conducive to vaccine hesitancy [24], However, attitudes may be more important, albeit more challenging to influence. In a previous study looking at the general US population, better knowledge about the vaccine and less acceptance of conspiracy theories were associated with higher COVID-19 vaccine acceptance [25], while education level was not consistently associated with believing vaccine misinformation across different countries [26]. Research in social psychology has provided strong evidence that better knowledge alone does not lead to greater motivation for behavioural change, but that changing attitudes may impact behaviour [27,28]. From a social marketing perspective, information should be presented in a way that positively influences attitudes, for example by informing about high vaccine coverage among the target group in other countries, rather than mentioning local insufficient coverage.

There is a considerable body of evidence on factors that influence COVID-19 vaccine acceptance and uptake among HCWs [29]. Other studies on COVID-19 vaccine acceptance by HCWs in Europe underpin the importance of trust and confidence [30-32]. We found a high overall capacity of this KA-7C questionnaire to explain variation in COVID-19 vaccine intention among healthcare and welfare sector workers. In comparison, socio-demographic differences explained a smaller proportion of variation (14%), which is surprising given the observation that vaccine coverage against influenza and against COVID-19 consistently differs substantially between socio-demographic and professional groups. A separate analysis will address the capacity of the KA-7C questionnaire to explain these variations between professional categories. Further psychometric analysis is required to validate the questionnaire and model structure with social conformism and confidence in systems as psychological antecedents.

Our study has some limitations. First, the study evaluates COVID-19 vaccine intention, but not eventual uptake. A considerable gap between vaccine intention and uptake exists [33], but looking at the factors that influence intention can at least contribute to explaining the thought processes that inform health decision-making as suggested in the Health Belief Model [33], the COM-B model [34] and the Theory of Planned Behaviour [35]. Second, for some knowledge questions we used relatively unspecific words, such as easily and frequently, which may not allow for the exact knowledge to be measured. However, more detailed estimates would probably have been difficult to expect apart from scientist HCWs and for some items (e.g. risk of long COVID-19) no precise estimate was available at the time of the survey. Third, data collection took place at the start of the COVID-19 vaccine campaign in France, during a period of constant communication of new information regarding vaccine efficacy and safety. However, our final model adjusted for the periods of survey participation such that the identified psychological antecedents should be independent of such trends. Finally, our results are limited to healthcare and welfare sector workers in France willing to participate in an online survey, while the relative importance of 7C item groups for COVID-19 vaccine intention may be different among non-participating healthcare and welfare sector workers, other population groups and other countries. Also, while the importance of social conformism and confidence in systems have been described for other vaccinations including influenza and childhood vaccinations, it likely has been exacerbated by the epidemic situation and may be lower in other contexts and with other vaccinations.

## Conclusions

Our study provides evidence that social conformism and confidence in systems are distinct psychological antecedents of vaccination and that attitude items play a larger role than knowledge in explaining vaccine intention. It may be worth considering social conformism and confidence in systems for more targeted vaccine promotion, although this would make the task more complex: a more detailed understanding of social influences and of opinions about working conditions and politics among subgroups is required. A first step could be insisting on the fact that vaccine decision among HCWs is a professional question to be addressed by occupational health, apart from private social environment on one side, and political claims on the other.

In January 2022, the COVID-19 vaccination mandate in France was extended to a booster dose. Our findings can help improve COVID-19 vaccine acceptance among French healthcare and welfare sector workers in the perspective of a long-term strategy beyond the mandate, but also in other population groups, for other vaccines and in other countries.

## Supplementary Data

311000 Supplementary Material
