# Supplementary material for: Social conformism and confidence in systems as additional psychological antecedents of vaccination: a survey to explain intention for COVID-19 vaccination among healthcare and welfare sector workers, France, December 2020 to February 2021
Source: Euro Surveill. 2022 Apr 28;27(17):2100617. doi: 10.2807/1560-7917.ES.2022.27.17.2100617 (PMC9052769; doi:10.2807/1560-7917.ES.2022.27.17.2100617)
Supplement: Supplementary Material [file 21-00617_MUELLER_Supplement.pdf]

This supplementary material is hosted by *Eurosurveillance* as supporting information alongside the article **Social conformism and confidence in systems as additional psychological antecedents of vaccination: a survey to explain intention for COVID-19 vaccination among healthcare and welfare sector workers, France, December 2020 to February 2021,** on behalf of the authors, who remain responsible for the accuracy and appropriateness of the content. The same standards for ethics, copyright, attributions and permissions as for the article apply. Supplements are not edited by *Eurosurveillance* and the journal is not responsible for the maintenance of any links or email addresses provided therein.

Supplementary Table S1. Full KA-7C COVID-19 vaccine intention questionnaire in French with English Translations asked to healthcare and welfare sector workers in France, December 2020 – February 2021.

French	English	Short name	Knowledge or attitude item	***Incorrect (0 points)***	***DNK (1 points)***	***Correct (2 points)***
1. J'ai peur d'avoir un effet secondaire grave après une vaccin.	I am afraid of having a severe side effect from the vaccination.	No fear of side effect	A	NA	NA	NA
2. Des considérations économiques pourraient conduire à une recommandation de vaccins insuffisamment évalués.	Economic considerations could lead to a recommendation of insufficiently evaluated vaccines.	Economic reasons for insufficiently evaluated vaccines	A	NA	NA	NA
3. J'ai peur de faire une forme grave de Covid-19.	I am afraid of getting a severe form of COVID-19.	Fear of severe form of COVID-19	A	NA	NA	NA
4. En pratique, il me sera difficile de me faire vacciner.	In practice, it will be difficult for me to get the vaccine.	Difficult access to vaccine	A	NA	NA	NA
5. Je pense que la vaccination contre la Covid-19 aura plus de bénéfices que de risques pour moi.	I think that vaccination against COVID-19 will have more benefits than risks for me.	More benefits than risks with vaccine	A	NA	NA	NA
6. Se faire vacciner sera aussi une action collective pour arrêter la crise liée à l'épidémie.	Getting vaccinated will also be a collective action to stop the crisis caused by the epidemic.	Collective action to stop the epidemic	A	NA	NA	NA
7. Si mon employeur m'incite à me faire vacciner, cela …	If my employer encourages me to get vaccinated...	Employer influence	A	NA	NA	NA
8. Dans votre entourage professionnel, comment décririez-vous l'opinion majoritaire envers la vaccination COVID-19?	In your professional environment, how would you describe the majority opinion towards COVID-19 vaccination?	Professional environment opinion	A	NA	NA	NA

9. Dans votre entourage familial et amical, comment décririez-vous l'opinion majoritaire envers la vaccination Covid-19?	In your familial and personal environment, how would you describe the majority opinion towards COVID-19 vaccination?	Family environment opinion	A	NA	NA	NA
Les affirmations suivantes expliquent-elles la rapidité inhabituelle du développement des vaccins Covid-19 ?	*The next affirmations explain the unusually rapid development of COVID-19 vaccines?*	NA	NA	NA	NA	NA
10. Avec des nouvelles technologies génétiques, on peut facilement formuler de nouveaux vaccins.	With new genetic technology new vaccines can easily be developed.	Genetic technology (easily develop new vaccines)	K	FALSE	DNK	TRUE
11. La gravité de l'épidémie nécessite de disposer rapidement d'un vaccin.	The gravity of the epidemic requires making vaccines quickly available.	Severity of epidemic	K	FALSE	DNK	TRUE
12. Des étapes d'évaluation (de contrôle) des nouveaux vaccins ont été supprimées à cause de la situation épidémique.	Some stages of vaccine development (testing) have been skipped due to the epidemic emergency.	Skip control steps in vaccine development	K	TRUE	DNK	FALSE
13. En France, qui peut signaler un effet secondaire après vaccination	In France, who can report a side effect after vaccination?	Report side effects	K	Only Doctors	All health professio nals	Both professiona ls & patients
Les affirmations suivantes sont-elles vrai ou faux?	*The next affirmations are true or false?*					
14. La sécurité des vaccins est surveillée non seulement au niveau national, mais aussi de façon collaborative entre les pays d'Europe	The security of vaccines is monitored not only at the national level, but also in collaboration between European countries	EU monitoring vaccines	K	FALSE	DNK	TRUE
15. Les effets secondaires graves peuvent se déclarer plus de six mois après la vaccination.	Severe side effects could appear in a person beyond 6 months after vaccination.	Severe side effects beyond 6 months	K	TRUE	DNK	FALSE
Pour la fin d'année 2020, indiquez pour les situations suivantes le niveau de risque de s'infecter avec le virus Sars-CoV-2.	*For the end of the year 2020, indicate for the next situations the level of risk of infection with the SARS-COV-2 virus.*					
16. Prendre une pause déjeuner ou un apéritif entre collègues soignants dans le service.	Taking a lunch break or a drink with colleagues in the service.	Lunch Risk	K	Low Risk	Medium Risk	High Risk

17. Toucher du matériel (ordinateur, téléphones) sans réaliser l'hygiène des mains.	Touching materials (computer, phone) without applying hand hygiene protocols.	Touch Risk	K	Low	NA	Medium/High Risk
18. En cas d'accident d'exposition au sang.	In case of accidental exposure to blood.	Blood Risk	K	High Risk	Medium Risk	Low Risk
19. Assister à un évènement festif d'environ 30 personnes.	Assisting in a festive event of 30 people.	Event Risk	K	Low Risk	Medium Risk	High Risk
20. L'obésité est un facteur de risque de forme grave à tout âge	Obesity is a risk factor at all ages.	Obesity Risk	K	FALSE	DNK	TRUE
21. Le facteur de risque principal d'une forme grave est l'âge à partir de 50-60 ans.	The principal risk factor of a severe form of COVID is age beyond 50-60 years.	Age Risk	K	FALSE	DNK	TRUE
22. Des symptômes prolongés (fatigue, trouble d'odorat, anxiété) sont fréquemment rapportés.	Prolonged symptoms (fatigue, trouble smelling, anxiety) are frequently reported.	Long COVID-19	K	FALSE	DNK	TRUE
23. A votre avis, pour une personne de 50 ans, le risque d'admission en réanimation ou de décès suite à une COVID-19 est d'environ.	In your opinion, someone over 50 years old has the risk of being admitted into the ICU.	ICU Risk for over 50	K	0.10%	DNK	1% & 10 %
Concernant les vaccins contre la COVID-19 les plus avancés (en voie d'autorisation), les données scientifiques montrent que …	*Concerning vaccines against COVID-19, the most. advanced (in the process of authorization), scientific data shows that...*	NA	NA	NA	NA	NA
24. … l'efficacité de ces vaccins est plus élevée que celle des vaccins contre la grippe.	The efficacy of getting the vaccine for COVID-19 is higher than for the flu vaccine.	Higher efficacy than flu vaccines	K	FALSE	DNK	TRUE
25. … pour les personnes avec facteur de risque, ces vaccins ont plus des bénéfices que de risques dans la situation épidémie actuelle.	For a person with risk factors, these vaccines have more benefits than risks in the current epidemic situation.	More benefits than risks for people with risk factor	K	FALSE	DNK	TRUE
26. … ces vaccins bloquent la transmission du virus à l'entourage en cas d'infection.	The vaccine blocks transmission of the virus to those around you in case of infection.	Vaccine blocks transmission if infected	K	TRUE	DNK	FALSE
27. ... il faudra deux injections pour être vacciné(e).	It is necessary to have 2 injections to be immunised.	Two doses needed	K	FALSE	DNK	TRUE

28. Connaissez-vous le pourcentage approximatif des soignants en France ayant l'intention de se faire vacciner contre la COVID-19, selon des sondages récents?	Do you know the approximate percentage of healthcare workers who intend to get vaccinated?	Vaccine coverage among HCWs	K	30%	DNK	60% & 90%
29. Sur une échelle de 0 à 10 : Globalement, l'épidémie de Coronavirus (COVID-19) en France vous inquiète-t-elle ?	On a scale from 0 to 10: Globally, the coronavirus (COVID-19) epidemic in France worries you?	Concern about the COVID-19 epidemic	A	NA	NA	NA
30. Sur une échelle de 0 à 10, quelle confiance accordez-vous aux autorités pour gérer la crise sanitaire et économique liée à la COVID-19?	On a scale from 0 to 10: How much confidence do you have in the authorities for managing the public health and economic crisis caused by COVID-19?	Confidence in crisis management	A	NA	NA	NA

A: attitude; K: knowledge

NA: not applicable

Supplementary Table S2. Distribution of responses to KA-7C items by intention to get vaccinated (Yes vs No/DNK), among French healthcare and welfare sector workers at the start of the COVID-19 vaccination campaign, 18 December 2020–1 February 2021 (n = 5234).

KA-7C [a]		COVID-19 Intention No/DNK		Yes		Bivariate (Yes vs No/DNK)	Individual item models adjusting for determinants [b] (Yes vs No/DNK)
		N	%	N	%	OR (95% CI)	OR (95% CI)
**Confidence in Vaccine**							
I am afraid of having a severe side effect of vaccination.	Disagree	372	14.6	2172	85.4	4.51 (3.81 - 5.34)	3.63 (3.04 - 4.33)
	Undecided	418	43.6	541	56.4	0.18 (0.15 - 0.22)	0.21 (0.17 - 0.25)
	Agree	1401	80.9	330	19.1	ref	ref
With new genetic technology new vaccines can easily be developed.	False (i)	409	75.7	131	24.3	ref	ref
	DNK	1068	56.0	840	44.0	2.46 (1.98 - 3.05)[b]	2.54 (2.00 - 3.21)
	True (c)	714	25.6	2072	74.4	9.06 (7.31 - 11.23)	7.58 (6.01 - 9.58)
In France, who can report a side effect after vaccination?	Only Doctors (i)	242	49.7	245	50.3	ref	ref
	All Health Professionals	217	37.4	363	62.6	1.65 (1.29 - 2.11)	1.19 (0.97 - 1.46)
	Everyone (c)	1732	41.6	2435	58.4	1.39 (1.15 - 1.68)	1.16 (0.88 - 1.52)
The security of vaccines is monitored not only at the national level, but also in collaboration between European countries.	False (i)	79	85.9	13	14.1	ref	ref
	DNK	596	69.7	259	30.3	2.64 (1.44 - 4.83)	2.70 (1.41 - 5.16)
	True (c)	1516	35.4	2771	64.6	11.11 (6.16 - 20.04)	10.63 (5.65 - 20.02)
Severe side effects could appear in a person beyond 6 months after vaccination.	False (c)	179	18.8	773	81.2	6.11 (5.05 - 7.38)	1.16 (0.88 - 1.52)
	DNK	1028	39.5	1574	60.5	2.16 (1.91 - 2.45)	1.22 (0.96 - 1.54)
	True (i)	984	58.6	696	41.4	ref	ref
**Confidence in Systems**							
Economic considerations could lead to a	Disagree	494	25.7	1432	74.4	ref	ref
	Undecided	679	41.5	956	58.5	0.49 (0.42 - 0.56)	0.26 (0.22 - 0.30)

recommendation of insufficiently evaluated vaccines.	Agree	1018	60.9	655	39.2	0.22 (0.19 - 0.26)	0.64 (0.55 - 0.75)
If my employer encourages me to get vaccinated, this …	Dissuades me	247	90.2	27	9.9	ref	ref
	Has no effect	1695	49.7	1714	50.3	9.25 (6.18 - 13.84)	7.61 (4.97 – 11.63)
	Motivates me	249	16.1	1302	84.0	47.83 (31.44 - 72.79)	45.05 (28.90 – 70.22)
Some stages of vaccine development (testing) have been skipped due to the epidemic emergency.	False (c)	721	75.2	238	524.8	14.07 (11.73 - 16.89)	13.55 (11.11 - 16.53)
	DNK	1071	52.9	952	47.1	2.69 (2.27 - 3.19)	3.34 (2.76 - 4.03)
	True (i)	399	17.7	1853	82.3	ref	ref
Confidence in crisis management	Low	917	64.4	506	35.6	ref	ref
	Medium	951	43.9	1217	56.1	2.32 (2.02 - 2.66)	2.69 (2.30 - 3.14)
	High	323	19.7	1320	80.3	7.41 (6.29 - 8.72)	7.81 (6.51 - 9.37)
**Complacency**							
I am afraid of getting a severe form of COVID-19.	Disagree	1201	45.6	1432	54.4	ref	ref
	Undecided	488	39.9	734	60.1	1.26 (1.10 - 1.45)	1.33 (1.14 - 1.55)
	Agree	502	36.4	877	63.6	1.47 (1.28 - 1.68)	1.60 (1.38 - 1.86)
The gravity of the epidemic requires making vaccines quickly available.	False (i)	331	80.5	80	19.5	ref	ref
	DNK	387	75.4	126	24.6	1.35 (0.98 - 1.85)	1.32 (0.94 - 1.87)
	True (c)	1473	34.2	2837	65.8	7.97 (6.19 - 10.25)	7.40 (5.63 - 9.74)
Taking a lunch break or a drink with colleagues in the service.	Low Risk (i)	169	63.1	99	37.0	ref	ref
	Med Risk	641	47.8	700	52.2	1.86 (1.42 - 2.44)	1.81 (1.34 - 2.46)
	High Risk (c)	1381	38.1	2244	61.9	2.77 (2.14 - 3.59)	2.70 (2.02 - 3.62)
Touching materials (computer, phone) without applying hand hygiene protocols.	Low Risk (i)	272	38.6	433	61.4	ref	ref
	Med Risk (c)	772	38.1	1255	61.9	1.02 (0.86 - 1.21)	1.26 (1.03 - 1.53)
	High Risk (c)	1147	45.8	1355	54.2	0.74 (0.63 - 0.88)	1.02 (0.84 - 1.24)
In case of accidental exposure to blood.	Low Risk (c)	1465	38.6	2326	61.4	1.66 (1.42 - 1.94)	1.30 (1.09 - 1.54)
	Med Risk	340	49.5	347	50.5	1.06 (0.87 - 1.31)	1.06 (0.85 - 1.33)
	High Risk (i)	386	51.1	370	48.9	ref	ref

Assisting in a festive event of 30 people.	Low Risk (i)	53	73.6	19	26.4	ref	ref
	Med Risk	189	59.4	129	40.6	1.90 (1.08 - 3.37)	2.30 (1.22 - 4.34)
	High Risk (c)	1949	40.2	2895	59.8	4.14 (2.45 - 7.02)	5.71 (3.17 - 10.27)
Obesity is a risk factor at all ages.	False (i)	28	53.9	24	46.2	ref	ref
	DNK	71	67.6	34	32.4	0.56 (0.28 - 1.10)	0.74 (0.35 - 1.58)
	True (c)	2092	41.2	2985	58.8	1.66 (0.96 - 2.88)	1.64 (0.88 - 3.03)
The principal risk factor of a severe form of COVID is age beyond 50-60 years.	False (i)	922	44.2	425	20.4	ref	ref
	DNK	236	50.0	115	24.4	0.79 (0.65, 0.97)	0.87 (0.70 - 1.09)
	True (c)	1033	38.6	498	18.6	1.26 (1.12 - 1.41)	1.22 (1.07 - 1.39)
Prolonged symptoms (fatigue, trouble smelling, anxiety) are frequently reported.	False (i)	29	26.9	79	73.2	ref	ref
	DNK	96	52.8	86	47.3	0.33 (0.20 - 0.55)	0.44 (0.25 - 0.78)
	True (c)	2066	41.8	2878	58.2	0.51 (0.33 - 0.79)	0.68 (0.42 - 1.09)
Which is the risk for someone 50 years or older, of being admitted to the ICU or dying in case of COVID-19?	0.1% (i)	342	43.6	443	56.4	ref	ref
	DNK	732	44.1	929	55.9	0.98 (0.83 - 1.16)	1.28 (1.05 - 1.54)
	1% & 10% (c)	1117	40.1	1671	59.9	1.15 (0.98 - 1.36)	1.41 (1.18 - 1.69)
The efficacy of vaccines against COVID-19 is higher than for the flu vaccine. [c]	False (i)	462	60.9	297	39.1	ref	ref
	DNK	1301	50.7	1265	49.3	1.51 (1.28 - 1.78)	1.51 (1.27 - 1.81)
	True (c)	428	22.4	1481	77.6	5.38 (4.49 - 6.45)	3.85 (3.17 - 4.69)
Concern about epidemic	Low	344	63.9	194	36.1	ref	ref
	Medium	793	50.2	788	49.8	1.76 (1.44 - 2.16)	0.96 (0.64 - 1.43)
	High	1054	33.8	2061	66.2	3.47 (2.86 - 4.20)	1.26 (0.84 - 1.88)
**Convenience**							
In practice, it will be difficult for me to get vaccinated.	Disagree	1382	36.2	2433	63.8	ref	ref
	Undecided	436	57.0	329	43.0	0.43 (0.36 - 0.50)	2.13 (1.70 - 2.66)
	Agree	373	57.0	281	43.0	0.43 (0.36 - 0.51)	4.42 (3.66 - 5.49)
It is necessary to have 2 injections to be immunised. [c]	False (i)	98	61.6	61	38.4	ref	ref
	DNK	372	71.0	152	29.0	0.66 (0.45 - 0.95)	0.64 (0.43 - 0.95)
	True (c)	1721	37.8	2830	62.2	2.64 (1.90 - 3.66)	1.54 (1.08 - 2.19)
**Calculation**							

I think that vaccination against COVID-19 will have more benefits than risks for me.	Disagree	1040	89.2	126	10.8	ref	ref
	Undecided	841	74.0	295	26.0	2.90 (2.31 - 3.64)	2.95 (2.32 - 3.74)
	Agree	310	10.6	2622	89.4	69.81 (56.10 - 86.92)	59.87 (47.57 - 75.36)
For a person with risk factors, these vaccines have more benefits than risks in the current epidemic situation. [c]	False (i)	124	83.8	24	16.2	ref	ref
	DNK	700	80.0	175	20.0	1.29 (0.81 - 2.06)	1.16 (0.71 - 1.88)
	True (c)	1367	32.5	2844	67.5	10.75 (6.91 - 10.72)	6.94 (4.38 - 11.00)
**Collective Responsibility**							
Getting vaccinated will also be a collective action to stop the crisis caused by the epidemic.	Disagree	528	92.5	43	7.5	ref	ref
	Undecided	620	90.4	66	9.6	1.31 (0.88 - 1.95)	1.44 (0.94 - 2.20)
	Agree	1043	26.2	2934	73.8	34.54 (25.11 - 47.51)	37.16 (26.42 - 52.27)
The vaccine blocks transmission of the virus to those around you in case of infection. [c]	False (c)	1095	41.7	1531	58.3	0.76 (0.65 - 0.90)	0.63 (0.52 - 0.76)
	DNK	820	44.9	1007	55.1	0.67 (0.56 - 0.80)	0.62 (0.51 - 0.75)
	True (i)	276	35.3	505	64.7	ref	ref
**Social Conformism**							
In your professional environment, how would you describe the majority opinion towards COVID-19 vaccination?	Skeptical	1085	64.5	597	35.5	ref	ref
	Both skeptical and favorable	860	42.7	1155	57.3	2.44 (2.14 - 2.79)	1.98 (1.72 - 2.20)
	Favorable	246	16.0	1291	84.0	9.54 (8.05 - 11.29)	5.65 (4.70 - 6.80)
Among your family and friends, how would you describe the majority opinion towards COVID-19?	Skeptical	1283	72.4	490	27.6	ref	ref
	Both skeptical and favorable	701	42.4	952	57.6	3.56 (3.08 - 4.10)	2.87 (2.47 - 3.34)
	Favorable	207	11.5	1601	88.6	20.25 (16.94 - 24.20)	13.49 (11.19 - 16.26)
	30% (i)	906	52.0	837	48.0	ref	ref

Do you know the approximate percentage of healthcare workers who intend to get the vaccine?	DNK	937	45.4	1127	54.6	1.30 (1.15 - 1.48)	1.11 (0.97 - 1.28)
	60% & 90% (c)	348	24.4	1079	75.6	3.36 (2.88 - 3.91)	2.23 (1.88 - 2.64)
**Sociodemographic Characteristics**							
Age (years)	18 - 34	681	56.1	534	44.0	ref	NA
	35 - 49	932	44.6	1160	55.5	1.59 (1.38 - 1.83)	NA
	50+	578	30.0	1349	70.0	2.98 (2.56 - 3.46)	NA
Gender	Female	1889	46.0	2214	54.0	ref	NA
	Male	302	26.7	829	73.3	2.34 (2.03 - 2.71)	NA
Profession	Nurses	603	50.4	594	49.6	ref	NA
	Nurse Assistants	341	69.5	150	30.6	0.45 (0.36 - 0.56)	NA
	Other Paramedical	407	49.7	412	50.3	1.03 (0.86 - 1.23)	NA
	Bio-Medical Professional [d]	287	19.8	1162	80.2	4.11 (3.46 - 4.88)	NA
	Admin./technical	553	43.3	725	56.7	1.33 (1.14 - 1.56)	NA
Nursing home	No	1766	39.9	2663	60.1	ref	NA
	Yes	425	52.8	380	47.2	0.59 (0.51 - 0.69)	NA
Flu Vaccine 2019 – 2020	No	1460	59.4	731	26.3	ref	NA
	Yes	997	40.6	2046	73.7	4.10 (3.65 - 4.61)	NA
Study Period	1	1113	54.9	913	45.1	ref	NA
	2	574	35.5	1044	64.5	2.22 (1.94 - 2.54)	NA
	3	504	31.7	1086	68.3	2.63 (2.29 - 3.01)	NA

(c): correct knowledge item response; DNK: Does not know; (i): incorrect knowledge item response; OR: odds ratio; Worry about epidemic: Worry about Covid-19 epidemic in France

[a] For simplicity of presentation, the items were reduced from the original 5-point to a 3-point Likert scale.

[b] Full multivariable model adjusting for age group, sex, professional category, work at a nursing home, and period of study participation.

[c] These questions were introduced as follows: "For the most advanced COVID-19 vaccines (close to licensure), the scientific data show that …"

[d] Including physicians, midwives, dentists, pharmacists and biologists.
